# Selected Socio-Demographic and Occupational Factors of Burnout Syndrome in Nurses Employed in Medical Facilities in Małopolska—Preliminary Results

**DOI:** 10.3390/ijerph15102083

**Published:** 2018-09-21

**Authors:** Anna Nowacka, Anna Piskorz, Renata Wolfshaut-Wolak, Jadwiga Piątek, Agnieszka Gniadek

**Affiliations:** 1 Department of Nursing Management and Epidemiological Nursing, Faculty of Health Sciences, Jagiellonian University Medical College, ul. Kopernika 25, 31-501 Kraków, Poland; anna.piskorz@uj.edu.pl (A.P.); agnieszka.gniadek@uj.edu.pl (A.G.); 2 Department of Epidemiology and Population Research, Faculty of Health Sciences, Jagiellonian University Medical College, ul. Grzegórzecka 20, 31-531 Kraków, Poland; renata.wolfshaut-wolak@uj.edu.pl; 3 Department of Health Psychology, Faculty of Health Sciences, Jagiellonian University Medical College, ul. Kopernika 25, 31-501 Kraków, Poland; jadwiga.piatek@uj.edu.pl

**Keywords:** nurse, occupational burnout, socio-demographic factors

## Abstract

The study examined the relationship between socio-demographic and occupational factors and the level of occupational burnout using the dimensions of emotional exhaustion (EE), depersonalization (DEP), and personal accomplishment (PA). It examined 560 nurses working in hospitals and primary healthcare units. We used: Maslach Burnout Inventory and a questionnaire including socio-demographic (sex, age, marital status, education, parental status) and occupational (period of employment, workplace, managerial functions, additional employment) factors. An average respondent was 38.13 (SD = 10.16) and had a BA degree (56.0%). The respondents reported average values of the EE (22.8), a low level of DEP (Me = 6), and a low PA (27.63). Nurses working on the intensive care unit had a chance of a high level of DEP that was 75% lower (OR = 0.25, 95% CI = 0.13–0.50) than nurses working in conservative treatment units. Additional employment increased the risk of a high level of DEP (OR = 2.86, 95% CI = 1.70–4.84). The chance of low PA was 64% lower in the case of nurse managers (OR = 0.36, 95% CI = 0.13–0.998) than other nurses. Education, period of employment, additional employment, and managerial position had a significant influence on the level of occupational burnout. An analysis of nurses’ work overload and additional employment can be an interesting research area.

## 1. Introduction

Although occupational burnout has been a subject of research for thirty five years, it is still a popular and frequently diagnosed problem which affects employees of all social services. It is currently becoming a burning issue of public health in numerous countries. In 2007, it was included as an underspecified additional diagnostic term in the International Statistical Classification of Diseases and Related Health Problems (ICD-10) in the section referring to factors influencing health status and contact with health services. ICD diagnosis code Z73.0 defines this condition as “Burn-out” [1].

### Burnout

Maslach and Jackson define burnout as a result of prolonged stress at work characterized by emotional exhaustion, depersonalization, and dissatisfaction with one’s accomplishments, which is mainly diagnosed in professional staff of human service institutions, including the nursing profession. Occupational burnout is a sequential process starting with emotional exhaustion which leads to depersonalization and, finally, to lower satisfaction with personal achievements [2,3]. Emotional exhaustion is treated by a significant group of researchers as the key aspect of burnout [4].

Pines and Aronson defined burnout as “a state of physical, emotional and mental exhaustion caused by long term involvement in situations that are emotionally demanding” [5,6]. Schaufeli and Enzmann, in turn, understand burnout as “a persistent, negative, work-related state of mind in ‘normal’ individuals that is characterized mainly by exhaustion and accompanied by distress, a sense of reduced competence, decreased motivation, and the development of dysfunctional attitudes at work”. This condition develops gradually and results from discrepancy between intentions and the reality of the job [7].

According to Cherniss, occupational burnout can be treated from a cognitive-competence perspective as a phenomenon of learned helplessness in situations of struggling with persistent stress. While facing extremely difficult working conditions with an enormous overload, people are only able to move on if they are capable of changing and modifying these conditions or, alternatively, adapting to them. A stable and strong conviction about one’s competences is essential to maintain and support attempts to change the environment or to adapt to new situations. In the case of people with low self-efficacy, the situation is just the opposite as their reactions include dissatisfaction, withdrawal, and indifference. Their attitude can be described as apathetic and passive [8,9].

According to the majority of researchers examining the problem of burnout, depersonalization plays a role which is different from a causal role of the other dimensions. Following S. E. Hobfoll’s *COR-Theory of Conservation of Resources*, which focuses on four resources (i.e., objects, conditions, personal characteristics, and energy), burnout is a continuous process caused by an ongoing, usually low-level, loss of resources. According to the Conservation of Resources theory, a persistent threat to valued resources culminates in burnout and these resources could also be connected with job performance. The development of burnout can be described as a spiral of resource loss which obtains its dynamic within the nexus of work stress and unsuccessful coping with it. Those who are affected by burnout either find their resources threatened with loss, or actually lose resources, or fail to adequately gain fresh resources after a significant resource investment.

*COR Theory* explains that depersonalization is often treated as a strategy of counteracting the burnout syndrome. In the long term, however, depersonalization limits access to social support and, consequently, causes isolation and decreases personal resources. Personal resources are understood as individual predispositions which allow people to function efficiently, especially in difficult and demanding situations. The Conservation of Resources theory is a useful conceptual framework for defining the four resources that may either increase or reduce nurse burnout [10,11].

Both in Poland and abroad, numerous researches into burnout determinants are being carried out, often emphasizing their multidimensional character. The burnout process develops as an interaction between the work environment and personal features. The causes of burnout can be divided into three groups: individual factors (low self-assessment, insecurity, dependency, the sense of external control, low self-efficacy); interpersonal factors including, above all, nurse-patient relations (regular contact with people in difficult situations, concentrating on traumatic experience of other people); and organizational factors (wrong management system, low status of work in social hierarchy, shortage of human resources, low salaries inadequate to the effort, bad working conditions, interpersonal conflicts, competition, lack of trust, aggression) [12,13].

In fact, burnout is a costly problem for both organizations and employees because manifestations of burnout—including reductions in physical and psychological energy, insomnia, headache, fatigue, and depression—lead to an increase in absenteeism and turnover rates and, consequently, have negative effects on the quality of care [14,15,16,17].

In the majority of Polish hospitals, nurses are exposed to enormous strain resulting from psychophysical work, responsibility for patients’ life and health, lack of job satisfaction, low salaries, low status of their work in the social hierarchy, and job insecurity [18,19,20].

Among the factors responsible for developing a burnout syndrome, an important role is played by socio-demographic factors. The most common ones include: age, work experience, sex, marital status, parental status, education, workplace, managerial position, and additional employment [21,22,23,24,25].

The objective of this study was, thus, as follows: first, to assess the level of burnout in professionally active nurses and secondly, to examine the relation between the examined dimensions of occupational burnout and selected socio-demographic and occupational factors to better understand the importance of nurse leaders’ role in mitigating the impact of burnout and, consequently, reducing turnover rates.

## 2. Materials and Methods

### 2.1. Design

A cross-sectional, descriptive, correlational design was used to explore the influence of selected socio-demographic and occupational factors on nurse burnout.

#### 2.1.1. Sample, Setting and Data Collection

The study was carried out between April 2014 and July 2016 in seven medical facilities (including five hospitals and two primary healthcare units) in the Małopolska region. The study was approved by the Bioethics Committee of Jagiellonian University Medical College and the managers of the medical facilities involved. It had a multicenter, cross-sectional, and observational character. The criteria for participation in the research included: written consent for participation, nursing profession, and proper workplace (public medical facilities: hospital, primary healthcare unit). As many as 738 people met the criteria, 560 of whom (75.88% of those who met the criteria) were classified for the analysis.

#### 2.1.2. Measurement

The self-designed questionnaire used in the study consisted of two parts. The first part included questions regarding socio-demographic variables: sex, age, marital status, parental status, and education, as well as questions which dealt with occupational variables such as the period of employment, workplace, system of work, managerial positions, doing overtime, and additional employment. For the sake of statistical analysis, the respondents were divided into three groups depending on their education: secondary vocational education, higher vocational BA education, and higher MA education.

The second part consisted of MBI-HSS (*Maslach Burnout Inventory Human Services Survey*), which examined three aspects of occupational burnout: emotional exhaustion (EE), depersonalization (DEP), and personal accomplishment (PA). The questionnaire consists of 22 statements. Each of the statements is connected with one of three separate subscales. The emotional exhaustion subscale includes nine statements: “I feel emotionally drained from my work”, “I feel used up at the end of the workday”, “I feel fatigued when I get up in the morning and have to face another day on the job”, “Working with people all day is really a strain for me”, “I feel burned out from my work”, “I feel frustrated by my job”, ”I feel I’m working too hard on my job”, “Working with people directly puts too much stress on me”, and “I feel like I’m at the end of my rope”. The depersonalization subscale includes five statements: “I feel I treat some recipients as if they were impersonal objects”, “I’ve become more callous toward people since I took this job”, “I worry that this job is hardening me emotionally”, “I don’t really care what happens to some recipients”, and “I feel recipients blame me for some of their problems”. The personal achievement subscale includes eight statements: “I can easily understand how my recipients feel about things”, “I deal very effectively with the problems of my recipients”, “I feel I’m positively influencing other people’s lives through my work”, “I feel very energetic”, “I can easily create a relaxed atmosphere with my recipients”, “I feel exhilarated after working closely with my recipients”, “I have accomplished many worthwhile things in this job”, and “In my work, I deal with emotional problems very calmly”. The frequency with which the respondents experience the feelings or attitudes described by the statements is assessed on a seven-point scale. There are seven frequency options ranging from 0 to 6. They appear in the following order: 0—never, 1—a few times a year, 2—once a month or less, 3—a few times a month, 4—once a week, 5—a few times a week, 6—every day. The subscales’ results were analyzed in the following way: first, the average value was defined for each of the examined subscales and then, according to the results, the categories were determined for relevant levels on each subscale. The results were interpreted according to the following reference norms: for the emotional exhaustion subscale, a score of 27 and more meant a high level, an average level ranged from 17 to 26, and a score between 0 and 16 was interpreted as a low level. For the depersonalization subscale, a score of 13 or more meant a high level, 7–12 indicated an average level, and 0–6 represented a low level. For the personal achievement subscale, a score of 39 and more meant a high level, 32–38 indicated an average level, and 0–31 denoted a low level. High scores on emotional exhaustion and depersonalization subscales accompanied by low scores on the personal achievement subscale indicates occupational burnout [26]). The reliability and accuracy of this diagnostic tool have been confirmed in Polish research (Cronbach’s α-coefficient above 0.7) [27].

#### 2.1.3. Analysis

The distribution of quality variables was described by quoting the frequency of absolute and relative values. The distribution of quantity variables was described by quoting the average values and standard deviation or, in the case when the value distribution was different from a regular distribution, by quoting the median and interquartile range. The Shapiro-Wilk test was applied to confirm the variable’s congruence with a normal distribution.

The relation between occupational burnout subscales and age, as well as the period of employment, was examined by means of Spearman’s rank correlation coefficient. The relation between selected socio-demographic features, workplace conditions/character (ward, additional employment, position), and particular Maslach scale categories was examined by means of a *chi square test*.

Logistic regression, both one- and multidimensional was used, so as to take into account more than one variable and examine how the analyzed factors were associated with the incidence of high emotional exhaustion, high level of depersonalization, and low satisfaction with personal achievement. The study includes the models of one-dimensional logistic regression prepared for each analyzed factor separately and a full model presenting all analyzed variables. Moreover, the correlation between socio-demographic and occupational factors was also tested using a likelihood ratio test. Due to the lack of significant correlations, the results are not presented in this paper.

Because of the very strong correlation between the age and period of employment r = 0.93, *p* < 0.001, only the period of employment was included in the analysis in order to avoid a model with two independent variables with such a strong correlation. A sensitivity analysis was carried out in which the period of employment was replaced by the respondents’ age.

Statistical analyses were carried out with the application of IBM SPSS Software for Windows, Version 23,0 (Armonk, NY (USA): IMB Corp). The significance level was estimated as α = 0.05 for two-tailed tests.

## 3. Results

### 3.1. Demographic and Work Condition Variables

The study was carried out in a group of 560 male and female nurses working in hospitals and primary healthcare units in the Małopolska region. The group consisted of 546 women (97.50%) and 14 men (2.50%). The average age of examined nurses was 38.13 (SD = 10.16), 72.32% of respondents were married (n = 405), and 34.64% (n = 194) had at least one child. The majority of respondents had higher education with a BA degree (55.89%), (n = 313). Among the respondents, 5.71% (n = 32) worked in managerial positions. The workplaces included conservative treatment units 49.46%, (n = 277), surgical wards 26.25%, (n = 147), intensive care wards 20.71%, (n = 116), and health centers 3.39%, (n = 19). As many as 36.43% of respondents (n = 204) declared that they were in some additional employment. The data are presented in Table 1.

### 3.2. Nurse Burnout

According to MBI, the general rate of occupational burnout in the examined group of nurses reached X = 57.9 SD16.96. High emotional exhaustion was observed in 37.14% nurses (n = 208), (an average value of this subscale reached 22.9 SD = 11.68). As far as the depersonalization subscale is concerned, 20.89% of respondents (n = 117) were classified in the high level category (Me = 6, lower quartile = 2, upper quartile = 11). Almost two thirds of respondents were classified at a low level of the personal achievement subscale (an average value of this subscale reached 27.63, SD = 9.31). Average values of the examined subscales of occupational burnout pointed out: an average level of emotional exhaustion (X = 22.90, SD = 11.68), low depersonalization (Me = 6), and a low sense of personal achievement in respondents (X = 27.63, SD = 9.31) (Table 2).

Similarly to Maslach and Johnson’s study, which showed correlations between the three subscales, our study also showed a positive correlation between depersonalization and emotional exhaustion (R = 0.32) and slightly weaker negative correlations between personal accomplishment, emotional exhaustion, and depersonalization (R = −0.15, R = −0.11). A weak positive correlation might also be observed between age, emotional exhaustion, and personal accomplishment (R = 0.15, R = 0.09), and a very weak but statistically significant correlation was found between age and depersonalization (R = −0.08). The period of employment had a positive influence on emotional exhaustion and personal accomplishment (R = 0.16, R = 0.08) and a negative influence on depersonalization (R = −10) (Table 3).

There are significant correlations between these three dimensions of burnout; however, they are not very strong (R = 0.33, R = −0.15 and R = −0.11), which allows the authors to assume that these three factors are independent. Moreover, these three items were obtained using explanatory factor analysis with “varimax rotation”. Therefore, it could be assumed that these three factors are orthogonal.

The correlation between socio-demographic and occupational factors was also tested using a likelihood ratio test. Due to the lack of significant correlations, the results are not presented in this paper.

Table 4 presents the correlation between selected socio-demographic variables, work characteristics, and the level of emotional exhaustion, depersonalization, and lowered satisfaction with personal achievement.

A significant relation was observed between age, period of employment, workplace, and emotional exhaustion. The respondents diagnosed with higher emotional exhaustion were usually older, with a longer period of employment, and more likely to work in intensive care units than those who were characterized by low or average emotional exhaustion. Furthermore, a significant relation between education, workplace, and depersonalization was observed. The highest percentage of respondents with high depersonalization belonged to the group with a BA degree (23.96%) (n = 75), *p* = 0.045, and the lowest percentage of respondents with high depersonalization was observed among nurses with a secondary education (13.16%) (n = 15).

The biggest difference in the percentage of respondents classified at a high level of depersonalization was observed between nurses employed in intensive care units and those employed in primary healthcare units (21.24%), *p* = 0.0003.

Managerial functions within the nursing subsystem, as well as the workplace, were factors which differentiated in a significant way the sense of satisfaction with personal achievement. Experiencing dissatisfaction with personal achievement was almost twice as frequent in the case of respondents without a managerial position as in the case of those employed in a managerial position. The highest percentage of respondents with a lowered sense of personal achievement was observed among the nurses employed in intensive care units (75.00%) (n = 87), *p* = 0.02, and the lowest among the nurses employed in primary healthcare units (52.63%) (n = 10).

Burnout subscales were also subjected to a quantitative analysis (the data were not published). The analyzed variables which were not significant in subscales’ category analysis were not significant either when they were examined in a quantitative way. The only difference was the fact that parental status had a significant impact on emotional exhaustion. Higher emotional exhaustion was observed in respondents who had children (X = 25.7, SD = 11.47) compared to childless participants (X = 22.2, SD = 11.85), *p* = 0.004.

Table 5 presents the results of multidimensional models describing the relation between the examined socio-demographic factors and work characteristics, and high emotional exhaustion and depersonalization accompanied by a low sense of personal achievement.

### 3.3. Emotional Exhaustion

In a multidimensional model, the period of employment had a significant influence on high emotional exhaustion (OR = 1.05, 95% CI = 1.03–1.08). The chances of high emotional exhaustion were 8% higher when the period of employment was one year longer, which confirms the rule known from previous research on the relation between the intensity of nurses’ occupational burnout and an increase in the number of tasks they are given. However, no significant relation was observed between the type of employment unit and high emotional exhaustion (a significant relation in a one-dimensional model). As compared to those working in a conservative unit, the respondents employed on a surgical ward had a 36% higher chance of experiencing high emotional exhaustion (OR = 0.64); however, the examined relation did not reach the level of statistical significance (95% CI = 0.34–1.19).

### 3.4. Depersonalization

In the multidimensional model, the factors significantly connected with high depersonalization included the place of employment and additional employment. Working on an intensive care ward was a protective factor as the nurses who were working there had a 75% lower chance of high depersonalization (OR = 0.25, 95% CI = 0.13–0.50) compared to nurses working in conservative treatment units. On the other hand, additional employment increased the chances of high depersonalization (OR = 2.86, 95% CI = 1.70–4.84).

### 3.5. Lowered Sense of Personal Achievement

A significant relation was observed between education, managerial position, and a lowered sense of personal achievement. The respondents with a higher education were twice as likely to experience dissatisfaction with their personal achievement (OR = 2.26, 95% CI = 1.05–4.83) compared to those with a secondary education. Higher education increases the sense of one’s competencies, but does not necessarily involve the possibility of participating in making decisions which are important in particular work. This phenomenon was confirmed by the results of the study carried out on people employed in a managerial position. Managerial work which involves making independent decisions turned out to be a protective factor. The likelihood of a lowered sense of personal achievement was 64% lower in respondents working in a managerial position than in nurses not working in a managerial position (OR = 0.36, 95% CI = 0.13–0.998). In comparison to the one-dimensional model, no significant relation was observed between a lowered sense of personal achievement and the place of work.

## 4. Discussion

Occupational burnout is a matter of significant concern in nursing, affecting both individuals and organizations. For individual nurses, the neuroendocrine response provokes physiologic reactions that may, consequently, lead to illness [28]. In health care organizations, burnout may contribute to absenteeism and a high turnover rate, both of which decrease the quality of care [29]. Health Care units (hospitals in particular) are facing a workforce crisis. The demand for intense care services is increasing concurrently with changing career expectations among potential nurses and growing dissatisfaction among contemporary hospital nurses [30,31,32].

A large number of studies of occupational burnout among nurses can be seen as indicative of the importance of this problem, which, despite the passage of time, is still current and is becoming a burning issue of both public health and the organization of medical services. The studies are carried out both in Poland and abroad in groups of medical professionals with the application of numerous research tools [32,33]. In Poland, numerous studies of occupational burnout in nurses are carried out; however, the application of a variety of both standardized and the authors’ own research tools, as well as unrepresentative groups of respondents, make it difficult to compare study results. While working on the study and browsing scientific publications on the subject, the authors examined the results of research into occupational burnout syndrome in medical staff carried out with the application of MBI-HSS (*Maslach Burnout Inventory Human Services Survey*) in groups including or consisting solely of nurses. The analysis was focused on the authors’ own professional group because it was proved that the interpretation of results obtained from occupational burnout subscales was different in the case of nurses than in the case of other groups of medical professionals [34]. In the authors’ opinion, the studies carried out in a group of 300 or more people have the highest scientific value. It is a cut-off point for carrying out an optimal factor analysis [35].

In the authors’ own study, nurses constituted 4.5% of medical staff employed in health facilities in the Małopolska region. The group was highly feminized (97.5% of women vs. 2.5% of men). The average age was 38.13, which was lower than the average age of nurses in Poland, reaching 44.24. The structure of education (secondary education—20.36%, MA degree—23.75% and BA degree—55.89%) was also different from the nationwide structure for Polish nurses, with the results from 2014 listing 60.4% of nurses with a secondary education, 28.8% of nurses with an MA degree, and 10.8% of nurses with a BA degree [36]. These differences stem from the shortages in nursing staff and dynamic changes in the system of nursing education in Poland.

The professional role of a nurse consists of nursing, helping, educating, and accompanying people in health, illness, and disability. In their working environment, nurses are exposed to a number of stress-inducing factors connected with protecting and rescuing human life. Occupational burnout syndrome is a worrying consequence of stress and it may have a further negative impact on nurses, as well as their patients and co-workers [37,38].

The percentage of nurses with a high level of emotional exhaustion (37.14% of the respondents), high depersonalization (20.89%), and lowered sense of personal achievement (64.29%) was, in this study, similar or slightly higher than the results of other Polish and foreign studies [4,7,15,22,23,24,39,40,41,42]. The study carried out by the authors in 2000 and in 2002 with the application of Maslach Burnout Inventory in a group of 104 nurses working in medical facilities in Kraków detected lower percentages for the first two subscales of burnout. High emotional exhaustion was detected in 28.8% of respondents, high depersonalization in 11.5%, and a lowered sense of personal achievement in 97.1% [43,44]. Similarly to the authors’ own study, another study carried out by Aiken L. H. et al. in the state of Pennsylvania (USA) showed that almost half of the respondents (43.2%) suffered from high emotional exhaustion [45]. Conversely, comparative studies (RN4 CAST) carried out in 12 European countries and in the USA detected a varying level of occupational burnout in terms of emotional exhaustion measured according to Maslach Burnout Inventory. A high level of emotional exhaustion (EE) ranged from 10% in the Netherlands, 40% in Poland, and 34% in the USA, to 78% in Greece [46]. Aforementioned results obtained by Polish nurses on the emotional exhaustion subscale are similar to the ones obtained in our study. In M. Sobczak’s study carried out in a group of 361 nurses, the general rate of occupational burnout reached X = 42.71 SD 19.65 [47], whereas in our study, the burnout rate was higher and reached 57.9 SD 16.96. Particular aspects of occupational burnout in this study reached the following average values: emotional exhaustion (EE) X = 22.9 SD 11.68, lowered sense of personal achievement (PA) X = 27.6 SD 9.31, and depersonalization Me = 6.0 (no average value because of irregular distribution).

In several studies, a lower level of burnout is associated with the professional character of the working environment [47], social support [48], and structural and psychological empowerment [49,50,51,52,53,54]. On the other hand, a high level of burnout is linked to work overload [49,54], job dissatisfaction, [17,45,48], and a high turnover rate [55]. Some demographic characteristics including low education level, night shifts [39], and male gender [48], are also associated with a high level of burnout.

In the authors’ study, the period of employment was significantly correlated with emotional exhaustion, which was confirmed in some other research in a group consisting of more than 200 nurses [35,38]. On the other hand, the studies carried out by Demir et al. in a group of 333 nurses and by Al-Turki et al. in a group of 198 nurses in 2003, showed a negative correlation between the period of employment and emotional exhaustion [21,39]. What is worrying about the Polish healthcare system is the fact that fewer and fewer people join the nursing subsystem. According to OECD data from 2012, the Polish rate of the number of nurses responsible for direct healthcare was the lowest in Europe, reaching 5.4/1000 citizens, whereas in Switzerland, for example, it reached 16/1000 and in Germany, 11.3/1000 [56].

The period of employment of a so-called “typical nurse” is getting longer and longer and it is more and more common for nurses to look for some additional employment in their profession in several healthcare facilities. According to the Main Chamber of Nurses and Midwives of Poland, it is possible that if nurses worked in just one place, the healthcare system would be unable to provide services requiring nurses’ participation or provided solely by nurses. Exposure to a number of stressors at work accompanied by low professional prestige and a low salary has a negative impact on emotional exhaustion in this professional group. In the examined group of nurses, a significant correlation was found between high emotional exhaustion and period of employment, which may result from work overload and, consequently, may have a negative influence on the quality of care and patient’s safety [27,34]. The results of other studies described in relevant publications indicate higher occupational burnout in nurses working in intensive care units and surgical wards [41], which was not found in the authors’ study. In the authors’ study, in turn, in the multidimensional model of analysis, a significant negative correlation was observed between working in an intensive care unit and depersonalization, so working in an intensive care unit turned out to be a protective factor. Working in an intensive care unit is frequently considered more professional and, therefore, is treated by nurses as a reward. The studies by Wilczek-Rużyczka and Basińska prove that rewards might protect against occupational burnout [57,58].

Additional employment had a positive correlation with the level of depersonalization, which was not confirmed in other available publications. A positive correlation was found between a lowered sense of personal achievement and the level of education of examined nurses, which coincides with some other studies proving that people with a higher education are more likely to suffer from occupational burnout. The interpretation of this phenomenon is inconclusive and might be complicated because education is associated with other variables, for example, professional prestige, social status, a responsible and stressful job, and higher expectations regarding one’s profession [35].

In our study, a managerial position which involves making independent decisions turned out to be a protective factor. However, though less frequently, nurses in supervisory positions may also experience burnout [59]. Psychological empowerment, in turn, had a strong positive effect on job satisfaction and a strong negative correlation with job strain. Likewise, as their perception of empowerment increased, nurses reported less emotional exhaustion and depersonalization, along with a greater sense of personal accomplishment [60].

In other studies, there were no differences between so called ‘first-line’ nurses and nurses in management positions. One study was conducted in the United States and the other one in Canada. Investigators for the Canadian study examined burnout in a random sample of nurses in first-line (n = 202) and middle-management (n = 84) positions. Nurses in both groups reported high levels of emotional exhaustion and average job satisfaction. In the U.S. study, the investigators explored burnout among nurses (n = 78) from rural and urban hospitals in a southeastern state who held positions in middle-management and higher. Almost half of the respondents (49%) reported high levels of emotional exhaustion [49,61].

Both researchers and nurse leaders believe that making the work environment healthier will result in improvements concerning the recruitment and retention of nurses, higher job satisfaction among all health care staff, and a positive impact on patients, especially in the area of their safety [62]. Because empowerment is often viewed as a characteristic of how work environments are structured, it has strong implications for nurse managers’ behaviors. However, one study revealed an interpretive side to empowerment that derives from nurses’ perceptions of their personal effectiveness and success [63]. Additionally, there appears to be evidence that nurse managers experience empowerment in a way that mirrors staff nurses’ experiences. That is, nurse manager perception of structural empowerment influenced their sense of psychological empowerment, which, in turn, affected the extent to which they experienced burnout [64].

The term workplace empowerment refers to employees’ ability to access the resources, information, and support needed to perform their work and to gain the opportunity to learn and develop [65,66].

While discussing nurses’ work environment it is important to take into consideration factors which might facilitate the prevention of occupational burnout syndrome. They include educational activities, proper selection for the nursing profession, psychological support, realistic requirements, developing positive relations in the team, and proper organization of work [67].

This study raised the issue of burnout as a research priority, and further research is required to assess the unique impact of different factors on burnout. In addition, more clinical trial and intervention studies are suggested to develop programs to reduce work stress as a strategy for attracting nurses, improving quality, and achieving optimal organizational outcomes.

## 5. Conclusions

The present study suggests that demographic variables and nurses’ work conditions are significant factors as far as nurse burnout is concerned. The model of multidimensional analysis made it possible to assess the influence of socio-demographic factors on the level of occupational burnout in nurses. The analysis of collected data showed that the symptoms of occupational burnout in the form of emotional exhaustion, depersonalization, and low satisfaction with one’s accomplishments can be observed in the examined group of male and female nurses. Socio-demographic factors such as education, period of employment, additional employment, or working in a managerial position had a significant influence on the level of occupational burnout. The results suggest that nurse managers and policy makers should improve nursing work conditions using the following strategies: reduce nurses’ workload through appropriate staffing, improve access to information, distribute resources fairly, and provide professional development opportunities.

### 5.1. Początek Formularza

Preventive actions of burnout should take place on three levels: organization, individual-organizational, and individual. On the level of the organization: recruitment of new employees and employment taking into account predispositions to perform a specific job, learning new skills, gaining knowledge both professional and applicable in coping with stress, improving physical and psychosocial working conditions, improving communication and cooperation in the organization, improvement within job control, skills utilization, workload, and work safety. On the individual-organizational level: promoting support from superiors and colleagues, matching individual job opportunities and requirements, clarifying roles: the scope of activities and introducing a participatory management model. On the individual level: developing the skills of relaxation in situations of increased tension; self-improvement; learning to recognize and regulate one’s mental states; reducing irrational thinking through the involvement of cognitive processes; the ability to accept unpleasant experiences and modify them, preventing them and their control; physical activity; diet; lifestyle; and developing the ability to delegate, negotiate, set goals, and confront the possibility of using individual advice from specialists in solving professional and non-professional problems.

These approaches are expected to decrease nurse burnout and consequently contribute to ongoing efforts to reduce the nursing shortage and improve the quality of care provided. An analysis of nurses’ work overload and their taking on additional work would be an interesting research area in the context of increasing occupational burnout in Polish nurses.

### 5.2. Study Limitations and Implications

The study took into account selected socio-demographic and occupational factors influencing the incidence of a burnout syndrome. Although the results of this study are robust, the study has some limitations: One of the limitations which might have influenced the results of of our study was the period of research. It might have been the source of the observed differences in the factors influencing occupational burnout. The authors’ individual study is a cross-sectional pilot study and it does not examine changeability of the phenomenon. The authors are planning to increase the number of examined nurses and to take into account an analysis of personality factors, attitudes towards work, and variables connected with psycho-social working conditions and work overload.

## Figures and Tables

**Table 1:** Socio-demographic characteristics of the examined group of nurses.

Socio-Demographic Variables	n	%	Average	SD
Sex				
Female	546	97.5	-	-
Male	14	2.5	-	-
Age			38.13	10.16
Marital status				
Single	155	27.68	-	-
Married	405	72.32	-	-
Parental status			-	-
Yes	194	34.64	-	-
No	366	65.36	-	
Education			-	-
Secondary vocational education	114	20.36	-	-
Higher education—BA degree	313	55.89	-	-
Higher education—MA degree	133	23.75	-	-
Workplace			-	-
Conservative treatment units	277	49.46	-	-
Surgical wards	147	26.25	-	
Intensive care wards	116	20.71	-	-
Primary healthcare units	19	3.39	-	-
Managerial position			-	-
Yes	32	5.71	-	-
No	528	94.29	-	-
Additional employment			-	-
Yes	204	36.43	-	-
No	356	63.57	-	-

**Table 2:** The incidence of three dimensions of occupational burnout in the examined group of nurses.

Dimensions of Occupational Burnout According to Maslach	n	%	Me	Average	SD
Emotional exhaustion (EE)				22.90	11.68
Low	189	33.75	-	-	-
Average	163	29.11	-	-	-
High	208	37.14	-	-	-
Depersonalization (DEP)			6.0	-	
Low	300	53.57	-	-	-
Average	143	25.54	-	-	
High	117	20.89	-	-	-
Personal achievement (PA)				27.63	9.31
Low	360	64.29	-	-	-
Average	128	22.86	-	-	-
High	72	12.86	-	-	-
General MBI burnout rate				57.9	(16.96)

**Table 3:** The matrix correlation between dimensions of burnout and age and period of employment.

	Emotional Exhaustion (EE)	Depersonalization (DEP)	Personal Accomplishment (PA)
Emotional exhaustion (EE)	-		
Depersonalization (DEP)	0.32 ***	-	
Personal accomplishment (PA)	−0.15 ***	−0.11 ***	-
Age	0.15 ***	−0.08 **	0.09 *
Period of employment	0.16 ***	−0.10 *	0.08 *

Note: * *p* < 0.05, ** *p* < 0.01, *** *p* < 0.001.

**Table 4:** Relation between selected socio-demographic factors and work characteristics and the level of emotional exhaustion, depersonalization, and personal achievement.

Variable	Low or Average Level		Emotional Exhaustion					Depersonalization					Personal Achievement		
			High Level		*p*	Low or Average Level		High Level		*p*	High or Average Level		Low Level	%	*p*
Marital status															
single (n, %)	102	65.81%	53	34.19%		115	74.19%	40	25.81%		55	35.5%	100	64.52%	
in a relationship (n, %)	250	61.73%	155	38.27%	0.37	328	80.99%	77	19.01%	0.08	145	35.8%	260	64.20%	0.9
Age (X, SD)	37.11	10.06	39.88	10.12	0.002	38.41	9.99	37.11	10.77	0.220	39.24	10.60	37.53	9.87	0.06
Period of employment (Me, Q1/Q3)	14	2.0/22.0	19	4.5/25.0	0.0008	15	3.0/23.0	15	2.0/21.0	0.08	15	3.0/23.5	15	2.0/23.0	0.19
Parental status															
no (n, %)	142	62.83%	84	37.17%		169	74.78%	57	25.22%		73	32.30%	153	67.70%	
yes (n, %)	106	54.64%	88	45.36%	0.088	154	79.38%	40	20.62%	0.3	65	33.51%	129	66.49%	0.8
Education															
Secondary vocational (n, %)	67	58.77%	47	41.23%		99	86.84%	15	13.16%		50	43.86%	64	56.14%	
Higher—BA (n, %)	208	66.45%	105	33.55%	0.13	238	76.04%	75	23.96%	0.045	111	35.46%	202	64.54%	0.06
higher—MA (n, %)	77	57.89%	56	42.11%		106	79.70%	27	20.30%		39	29.32%	94	70.68%	
Managerial position															
no (n, %)	330	62.50%	198	37.50%		421	79.73%	107	20.27%		179	33.90%	349	66.10%	
yes (n, %)	22	68.75%	10	31.25%	0.5	22	68.75%	10	31.25%	0.14	21	65.63%	11	34.38%	<0.001
Employment place															
conservative treatment (n, %)	161	58.12%	116	41.88%		201	72.56%	76	27.44%		100	36.10%	177	63.90%	
surgical ward (n, %)	107	72.79%	40	27.21%		124	84.35%	23	15.65%		62	42.18%	85	57.82%	
intensive care (n, %)	70	60.34%	46	39.66%	0.002	104	89.66%	12	10.34%	0.0003	29	25.00%	87	75.00%	0.02
primary healthcare (n, %)	13	68.42%	6	31.58%		13	68.42%	6	31.58%		9	47.37%	10	52.63%	
Additional employment															
no (n, %)	221	62.08%	135	37.92%		289	81.18%	67	18.82%		119	33.43%	237	66.57%	
yes (n, %)	131	64.22%	73	35.78%	0.6	154	75.49%	50	24.51%	0.11	81	39.71%	123	60.29%	0.14

**Table 5:** Multidimensional relation between selected socio-demographic factors and work characteristics, and high emotional exhaustion and depersonalization accompanied by low sense of personal achievement.

	High Emotional Exhaustion			High Depersonalization			Low Sense of Personal Achievement		
Variables	OR	95% CI		OR	95% CI		OR	95% CI	
Marital status									
Single	1			1			1		
in a relationship	1.31	0.76	2.25	0.95	0.52	1.72	0.97	0.56	1.67
Parental status									
No	1.00			1.00			1.00		
Yes	0.66	0.37	1.18	0.99	0.54	1.84	1.15	0.63	2.08
Education									
secondary vocational				1.00					
Higher—BA degree	0.61	0.32	1.17	1.65	0.71	3.86	1.66	0.87	3.17
higher—MA degree	0.85	0.41	1.79	1.26	0.48	3.29	2.26	1.05	4.83
Period of employment	1.05	1.03	1.08	0.98	0.96	1.01	1.01	0.98	1.03
Managerial position									
No	1.00			1.00			1.00		
Yes	0.38	0.12	1.23	1.31	0.38	4.45	0.36	0.13	0.998
Employment place									
conservative treatment	1.00			1.00			1.00		
surgical ward	0.64	0.34	1.19	0.67	0.33	1.34	0.99	0.54	1.79
intensive care	0.85	0.52	1.37	0.25	0.13	0.50	1.58	0.94	2.64
primary healthcare	1.08	0.34	3.48	1.16	0.35	3.81	0.73	0.24	2.25
Additional employment									
No	1.00			1.00			1.00		
Yes	1.20	0.75	1.92	2.86	1.70	4.84	0.88	0.54	1.44
